# Enzymatic production of single-molecule FISH and RNA capture probes

**DOI:** 10.1261/rna.061184.117

**Published:** 2017-10

**Authors:** Imre Gaspar, Frank Wippich, Anne Ephrussi

**Affiliations:** Developmental Biology Unit, European Molecular Biology Laboratory (EMBL), Heidelberg 69117, Germany

**Keywords:** terminal deoxynucleotidyl transferase, dideoxy-UTP, oligonucleotide labeling, single-molecule FISH, RNA affinity purification

## Abstract

Arrays of singly labeled short oligonucleotides that hybridize to a specific target revolutionized RNA biology, enabling quantitative, single-molecule microscopy analysis and high-efficiency RNA/RNP capture. Here, we describe a simple and efficient method that allows flexible functionalization of inexpensive DNA oligonucleotides by different fluorescent dyes or biotin using terminal deoxynucleotidyl transferase and custom-made functional group conjugated dideoxy-UTP. We show that (i) all steps of the oligonucleotide labeling—including conjugation, enzymatic synthesis, and product purification—can be performed in a standard biology laboratory, (ii) the process yields >90%, often >95% labeled product with minimal carryover of impurities, and (iii) the oligonucleotides can be labeled with different dyes or biotin, allowing single-molecule FISH, RNA affinity purification, and Northern blot analysis to be performed.

## INTRODUCTION

The ability to quantify biomolecules in space and time in living matter has introduced a qualitative change in many fields of biology, allowing the building of accurate mathematical models that can test, validate, and refine hypotheses about biological processes. Quantitative imaging of RNA, for instance, allows precise measurement of transcript copy number within cells, subcellular compartments, and ultimately in single ribonucleoprotein (RNP) complexes (Weil et al. 2010; Gaspar and Ephrussi 2015). Such studies have identified heterogeneity at all these levels: between cells (Raj and van Oudenaarden 2008), subcellular regions (Medioni et al. 2012), and between individual RNPs. While some mRNPs contain only a single type and a single copy of an mRNA molecule, others may undergo dynamic homo- or heterotypic oligomerization, resulting in hundreds of copies of the mRNA within a single RNP particle (Little et al. 2015). Although today a wide variety of RNA detection techniques with single-molecule sensitivity exists (Gaspar and Ephrussi 2015), the pioneering techniques of single-molecule fluorescent in situ hybridization (smFISH) were based on an array of short oligonucleotides carrying a well-defined number of labels, thus reducing variance of the detected signal while maintaining a high signal-to-noise ratio (Femino et al. 1998; Raj et al. 2008).

Such arrays typically consist of 24–96 different probes: 18- to 22-nucleotide (nt)-long single-stranded DNA (ssDNA) molecules complementary to nonoverlapping segments of the target RNA, each carrying a single label (Raj et al. 2008). Due to the multiple probe molecules hybridizing to a single target, this design ensures a linearly amplified signal. As the vast majority (>90%) of the probes carry only a single covalently coupled fluorescent molecule, heterogeneity of signal due to labeling is minimal in the case of smFISH probes, in contrast to conventional FISH probes. Furthermore, the relatively large number of probes ensures reliable detection of virtually all transcripts found within the specimen.

Due to the strict design principles, these probes are synthesized chemically and then purified by analytical means, such as HPLC or PAGE isolation (Raj et al. 2008). The coupling of the fluorophore takes place before purification and the resulting probe or probe set is rather static, such that there is no simple means to change to a spectrally different fluorophore, and it is often difficult or impossible to alter the composition of the oligonucleotides in the set without restarting the entire synthesis process.

An alternative to precoupled fluorophore probe sets are functionalized oligos that can be chemically coupled to a given molecule by the experimenter. The most commonly used functionalization is a primary amine modification at either end of the oligonucleotides, which enables simple coupling of dye–succinimidyl (NHS)-ester conjugates. In this way, aliquots of the same probe set can be labeled differently depending on the experimental requirements. However, such custom labeling puts the burden of purification—i.e., removing the unconjugated fraction of the oligos and the free dye molecules—on the experimenter. Also, the reactivity of NHS esters decays rapidly as a function of storage time due to hydrolysis, which can result in unpredictable labeling and wasting of expensive reagents.

**FIGURE 1.**
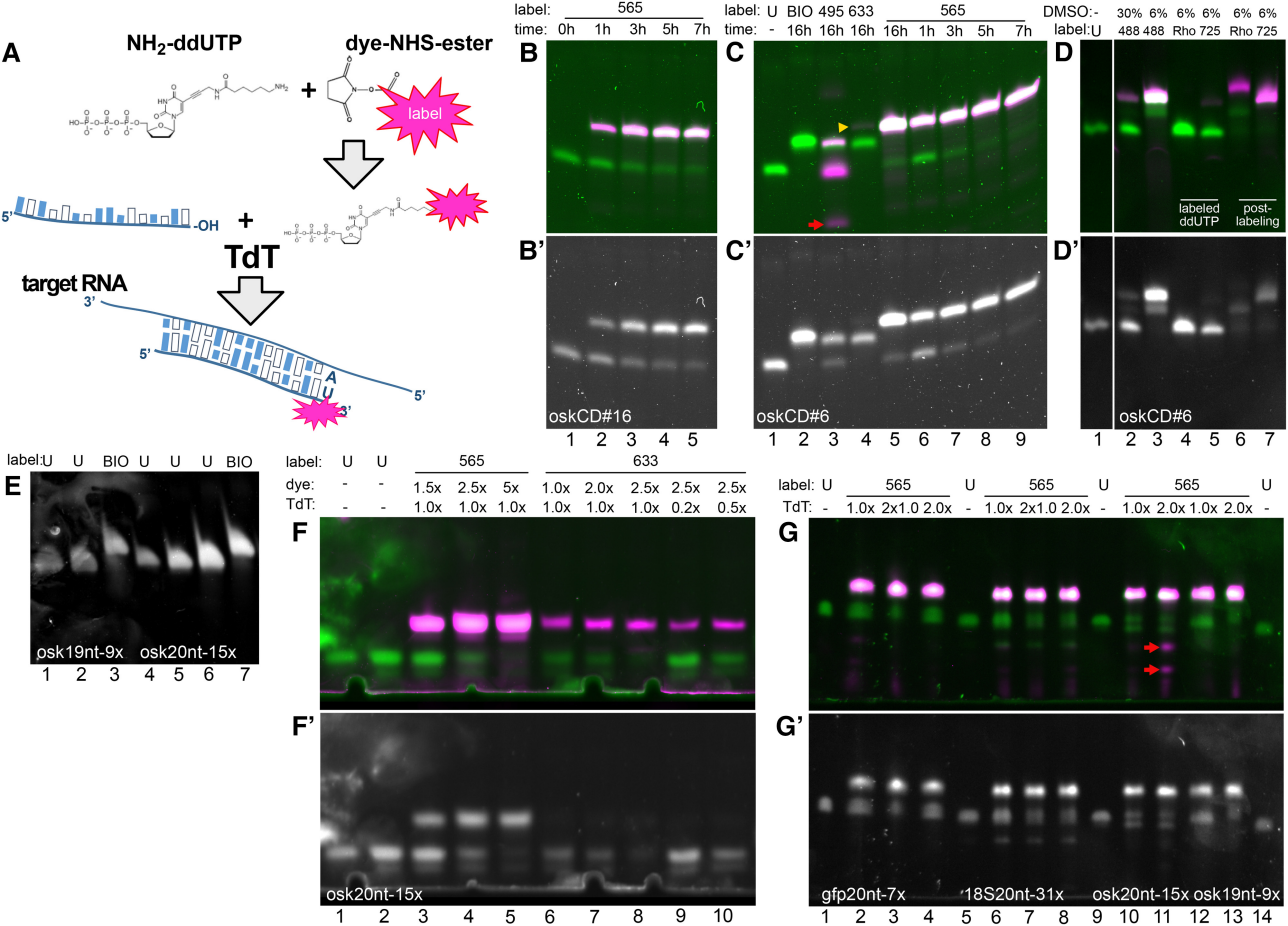
3′ Incorporation of labeled ddUTP by TdT. (*A*) Schematics of the enzymatic oligo labeling protocol. The volatile dye–NHS ester is reacted with NH_2_-ddUTP immediately upon reconstitution. The labeled, unpurified terminator nucleotide is then used in a TdT-mediated end-labeling reaction to label the 3′ end of individual oligonucleotides or oligonucleotide mixtures that can serve as smFISH or RNA affinity capture probes in downstream applications. (*B*,*B*′) oskCD#16 probe (lane *1*) labeled with Atto565–ddUTP (4× molar excess) for 1 h (lane *2*), 3 h (lane *3*), 5 h (lane *4*), and 7 h (lane *5*). Dye fluorescence is shown in magenta, SYBR GOLD staining is shown in green (*B*) and in gray (*B*′). Ninety-six picomoles of oligo/lane. (*C*,*C*′) oskCD#6 probe (lane *1*) labeled with biotin–ddUTP (threefold molar excess) (lane *2*), Atto495–ddUTP (fourfold molar excess) (lane *3*), Atto633–ddUTP (fourfold molar excess) (lane *4*), and Atto565–ddUTP (fourfold molar excess) (lane *5*) overnight (16 h). Red arrow (lane *3*) indicates carryover of free Atto495–ddUTP. Yellow arrowhead points to a minuscule amount of Atto633 labeled, fluorescent product in lane *4*. Time course labeling of oskCD#6 probe with Atto565–ddUTP (fourfold molar excess) for 1 h (lane *6*), 3 h (lane *7*), 5 h (lane *8*), and 7 h (lane *9*). Ninety-six picomoles of oligo/lane. (*D*,*D*′) oskCD#6 probe (lane *1*) labeled with Atto488–ddUTP (fourfold molar excess) in the presence of 30% (lane *2*) and 6% DMSO (lane *3*). oskCD#8 probe labeled directly with AttoRho14–ddUTP (lane *4*) and Atto725–ddUTP (lane *5*) (fourfold molar excess) or with unconjugated NH2–ddUTP reacted to AttoRho14–NHS ester (lane *6*) or Atto725–NHS ester (lane *7*) subsequently. Ninety-six picomoles of oligo/lane. (*E*) osk19nt-9× probe mixture (lane *1*, 12 pmol; lane *2*, 24 pmol) labeled with biotin–ddUTP (threefold molar excess) (lane *3*, 24 pmol). osk20nt-15× probe mixture (lane *4*, 6 pmol; lane *5*, 12 pmol; lane *6*, 24 pmol) labeled with biotin–ddUTP (threefold molar excess) (lane *7*, 24 pmol). (*F*,*F*′) osk20nt-15× probe mixture (lane *1*, 6 pmol; lane *2*, 9 pmol) labeled with Atto565–ddUTP (lane *3*, 1.5-fold; lane *4*, 2.5-fold; lane *5*, fivefold molar excess) or with Atto633–ddUTP (lane *6*, 1.5-fold; lane *7*, twofold; lanes *8*–*10*, 2.5-fold molar excess). Lanes *9* and *10* show results of labeling performed with reduced amounts of TdT enzyme (lane *9*, 0.2-fold; lane *10*, 0.5-fold of standard TdT amount). Fifteen picomoles of oligo/lane (*3*–*10*). (*G*,*G*′) Atto565–ddUTP labeling (fivefold excess) of probe mixtures. gfp20nt-7× probe mixture (lane *1*) labeled using onefold (lanes *2*,*3*) or twofold (lane *4*) the standard TdT amount. 18S20nt-31× probe mixture (lane *5*) labeled using onefold (lanes *6*,*7*) or twofold (lane *8*) the standard TdT amount. osk20nt-15× probe mixture (lane *9*) labeled using onefold (lane *10*) or twofold (lane *11*) of standard TdT amount. osk19nt-9× probe mixture (lane *14*) labeled using onefold (lane *12*) or twofold (lane *13*) the standard TdT amount. Lanes *3* and *7* show results of relabeling of probe mixtures shown in lanes *2* and *6*, respectively. Three picomoles of oligo/lane (*1*,*5*,*9*,*14*) and 15 pmol oligo/lane (*2*–*4*,*6*–*8*,*10*–*13*). Red arrow (lane *11*) indicates carryover of free Atto565–ddUTP.

Here we present a simple, efficient, and flexible alternative to smFISH and RNA affinity purification (RAP) probe production that can be carried out in any laboratory using basic equipment. This method relies on the template-independent addition of dNTPs at the 3′ end of DNA by terminal deoxynucleotidyl transferase (TdT) (Motea and Berdis 2010). This enzyme could elongate 3′ ends indefinitely; however, the use of dideoxy nucleotides ensures that only a single labeled nucleotide is incorporated into each and almost every oligonucleotide molecule added to the reaction. Although the feasibility of such a direct labeling strategy was demonstrated previously for individual oligonucleotides (Jahn et al. 2011; Winz et al. 2015), here we show that it is possible to label sets of conventional PCR oligos using custom-made dye/biotin conjugated ddUTPs synthesized in a standard biology laboratory (Fig. 1A). The synthesis results in >90% (often >95%) of singly labeled probe molecules, which—after a simple precipitation based purification—are suitable for quantitative imaging with single-molecule sensitivity and for RAP. We describe the optimized conditions for incorporation of spectrally different Atto565–ddUTP and Atto633–ddUTP as well as biotin–ddUTP into ssDNA oligonucleotides.

## RESULTS

### Incorporation of unpurified fluorophore–ddUTP conjugates to the 3′ of oligonucleotides by TdT

To make enzymatic oligo end-labeling a technique readily available for most laboratories, we identified simplicity and cost efficiency—as well as synthesis efficiency—as the main criteria for such an assay. Therefore, we first tested whether TdT could incorporate unpurified Atto565–ddUTP, synthesized through conjugation of Atto565–NHS ester to NH_2_-ddUTP, into the 3′ end of single DNA oligonucleotides. We found that, given sufficient time (∼7 h), even relatively low amounts of the enzyme can yield near-qualitative labeling of both oskCD#16 and oskCD#6 oligos, as visualized upon denaturing 8 M urea-PAGE (Fig. 1B,C). The labeled fraction of the oligos yielded fluorescence in the red spectrum and, due to the addition of a nucleotide plus a bulky, positively charged dye, migrated more slowly during electrophoresis. This migratory retardation is large enough to distinguish incorporation of unlabeled NH_2_-ddUTP and dye-conjugated-ddUTP (Fig. 1; Supplemental Fig. S1). As NHS esters are known to lose reactivity rapidly in solution, we conjugated NH_2_-ddUTP with aliquots of Atto633 (twofold molar excess) and Atto488 (10-fold molar excess) that were several (5–7) months old. As shown in Figure 1C, lane 4 and Figure 1D, lane 3, virtually all the oligonucleotide was labeled (i.e., the band corresponding to the unlabeled nucleic acid disappeared); however, in addition to a fluorescent, slow migrating band (Fig. 1C, yellow arrowhead), a nonfluorescent product could be detected that reflects the incorporation of unlabeled NH_2_-ddUTP. Increased concentrations of DMSO (30 v/v%)—as a result of a large molar excess of the dye–NHS ester during conjugation—are detrimental to the synthesis (Fig. 1C, lane 2), hence the reaction must be diluted to bring the DMSO concentration below 6 v/v% (Fig. 1D, lane 3). The appearance of a middle band is an indicator of an incomplete conjugation reaction in the case of positively charged dyes (e.g., Atto488, Atto565, or Atto633). Unfortunately, this does not apply to negatively charged dyes, such as Atto495 or small, neutral compounds, such as biotin (Fig. 1C,E) or BDP-FL (Supplemental Fig. S1).

Other dyes, such as AttoRho14 and Atto725, on the other hand, were readily conjugated to primary amines, but the resulting dye–ddUTP product was not incorporated during the synthesis (Fig. 1D, lanes 4,5). Nevertheless, it was possible to use the NHS-ester of these dyes to conjugate NH2-ddUTP labeled oligos (Fig. 1D, lanes 6,7).

### Enzymatic production of smFISH and RNA capture probes

For efficient and relatively simple synthesis of smFISH and RNA capture probe sets, the assay should yield >90%, ideally >95% of labeled oligonucleotides (as offered by vendors of smFISH probes) with minimal contamination of the free dye–ddUTP conjugate. Also, the assay should be capable of labeling not only single oligos, but also pools of different oligonucleotides. To fulfill these criteria, we optimized the labeling reactions by varying some of the experimental conditions, for instance using different probe sets containing oligos of the same length (osk-19nt-9×, osk-20nt-15×, gfp-20nt-7×, 18S-20nt-31×). We found that efficient incorporation of the different ddUTP conjugates required slightly different conditions: A threefold excess of biotin–ddUTP and standard amounts of TdT were sufficient to label virtually all molecules of the probe sets (Fig. 1E). In the case of Atto633–ddUTP, we needed as little as a 2.5-fold excess (although, as a precaution, in our protocols we use a threefold excess of this nucleotide) and a standard amount of TdT to obtain near qualitative labeling (Fig. 1F). However, substandard amounts of the enzyme negatively impacted the synthesis (Fig. 1F, lanes 9–10). In the case of Atto565–ddUTP, we used a fivefold molar excess to obtain similar results (Fig. 1F). Although a standard amount of the enzyme worked sufficiently in the case of the osk-20nt-15× probe set, efficient labeling of other probe sets (e.g., 18S-20nt-31× or osk-19nt-9×) required twice as much enzyme as the standard (Fig. 1G). In the case of Atto633–ddUTP, threefold molar excess of the ddUTP conjugate and standard amounts of TdT worked for all other probe sets (data not shown). Efficient labeling of the gfp-20nt-7× probe set with Atto565–ddUTP turned out to be especially difficult, probably due to the intra- and intermolecular interactions of the individual oligonucleotides, which had a high melting temperature (*T*_m_) as a consequence of the GC richness of the *gfp* coding sequence. However, we found that subjecting the labeled oligo mixture to a second round of labeling (using standard amounts of TdT in both synthesis steps) could yield >90% labeled gfp-20nt-7× product (Fig. 1G, lanes 2,3,6,7).

**FIGURE 2.**
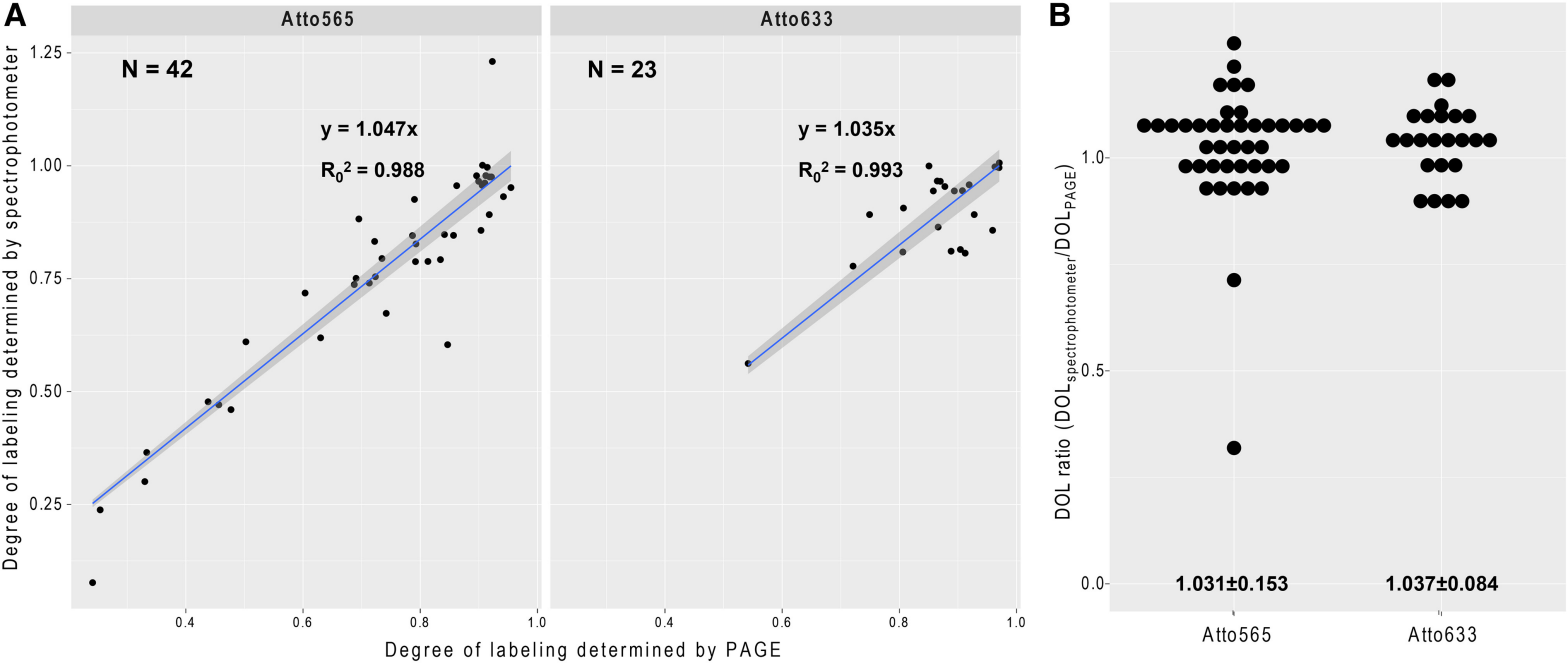
Determining degree of labeling. (*A*) Degree of labeling of isomeric probe mixtures (osk19nt-7×, osk20nt-15×, gfp-19nt-11×, gfp-20nt-7×, and 18S-20nt-31×) determined by PAGE densitometry and by spectrophotometry using Atto565– or Atto633–ddUTP. Correlation of the two analytical modalities was tested, slope and goodness of fit, as well as the number of measurements are indicated in the graphs. The intercept of the regression model was set to zero. (*B*) Ratio of spectroscopically and densitometrically determined degree of labeling. Mean ± SD are indicated *below* the graphs.

Although there are different ways to purify the oligos after the synthesis (e.g., gel filtration, HPLC, or PAGE purification), we found that for most of the dye–ddUTP conjugates—with the exception of Atto 495 (Fig. 1C, red arrow), Abberior 470 SX, Atto488, and AlexaFluor488 (Supplemental Fig. S1, red arrow)—a simple, linear acrylamide facilitated ethanol precipitation, and subsequent washes usually resulted in a clean product with little or no visible trace of the free dye or the dye–ddUTP conjugate (Fig. 1G, lane 11, red arrows). Such purification further simplifies the handling of these oligo mixtures and, starting with 1000 pmol of unlabeled oligo, the typical loss is 22.0 ± 12.9% (mean ± SD, *N* = 33). Another implication of the low carryover of fluorescent impurities is that the degree-of-labeling (DOL, i.e., the fraction of labeled oligos) determined by spectroscopy of the reconstituted oligo mixture is expected to correlate well with that measured by densitometry on PAGE. To test this hypothesis, we plotted the spectroscopic DOL as a function of densitometric DOL of the dye-labeled oligo mixtures and fitted the plots with a linear regression model (Fig. 2A). We found a great fit between our data and the linear model (*R*^2^ > 0.98) with a slope slightly above 1 for both Atto565 and Atto633 labeling, and in the vast majority of cases the ratio of the two DOL values was within 10% of the mean (Fig. 2B). This indicates that spectroscopy provides a good—although slightly overestimating—measure of the DOL of these enzymatically produced smFISH probes.

### Assaying performance of enzymatically produced smFISH probes

**FIGURE 3.**
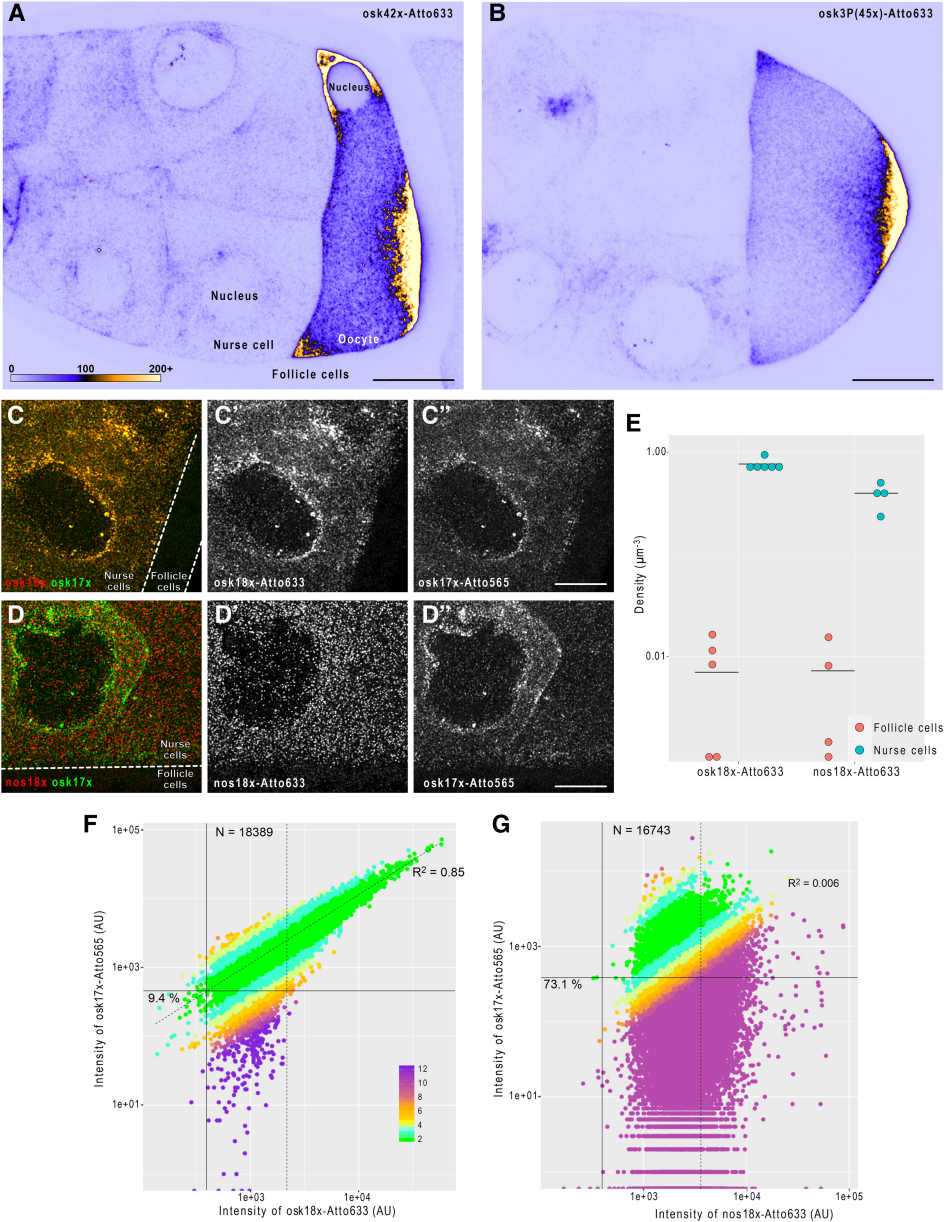
smFISH carried out using 3′ end labeled probe mixtures. (*A*,*B*) Fluorescent in situ hybridization of *oskar* mRNA in mid-oogenetic *Drosophila* egg chambers using a set of 42 enzymatically produced antisense oligonucleotides (*A*) or a set of 45 chemically labeled smFISH probes (*B* [Little et al. 2015]). Posterior poles, where *oskar* mRNA localizes, face toward the *right* of the panels. Sum intensity projections of 11 optical slices (a total 2 µm depth) of raw confocal images are shown. Pixels represent absolute photon counts (see panel *A* for key). Scale bars are 20 µm. (*C*–*C*″) smFISH of *oskar* mRNA in a developing *Drosophila* egg chamber using osk18×–Atto633 (red, *C*′) and osk17×–Atto565 (green, *C*″) probe sets. The two probes sets label *oskar* mRNA in an alternating fashion. (*D*–*D*″) smFISH of *oskar* and *nanos* mRNAs in a developing *Drosophila* egg chamber using nos18×–Atto633 (red, *D*′) and osk17×–Atto565 (green, *D*″) probe sets. (*C*–*D*″) The germline, which expresses *oskar* and *nanos* mRNAs (nurse cells) and the soma (follicle cells), is indicated. Scale bars are 10 µm. (*E*) Density of detected smFISH signal in the mRNA expressing (cyan, nurse cells) and nonexpressing (red, follicle cells) compartments of the egg chambers. *Horizontal* lines indicate the mean value of observations. (*F*,*G*) Intensity of osk17×–Atto565 probe sets (target channel) as a function of Atto633 signal intensity (reference channel) of smFISH objects detected by the fluorescence of osk18×–Atto633 (*F*) or nos18×–Atto633 (*G*) probe sets (reference channel). Percentage values represent the fraction of single mRNA containing smFISH objects that would have failed to be detected in the target channel (Atto 565) since their corresponding signal intensity falls below the estimated target channel detection threshold (solid *horizontal* line). This detection threshold corresponds to the product of the reference channel detection threshold (solid *vertical* line, 0.1th percentile of the Atto633 signal intensity distribution) and the slope of the fitted line (dashed line. The line was fitted to the nonsingle mRNA representing fraction of the population; goodness-of-fit indicated). smFISH objects with signal intensity lower than µ_1_ + 2σ_1_ of the smallest fitted Gaussian function (Supplemental Fig. S2) were considered to represent a single mRNA molecule (dashed *vertical* line). Numbers of these single copy mRNA objects are indicated in the graphs. Colors represent the relative fold difference of the observed and expected target channel (Atto565) signals (see panel *F* for key). The expected target channel (Atto565) signal is calculated as the product of the regression slope and the reference channel (Atto633) fluorescence.

smFISH probes are expected to detect even a single copy of their target mRNA with high sensitivity (low level of false negatives) and with high specificity (low level of false positives). To test the performance of the labeled probe sets, we chose the developing *Drosophila* egg chamber as a model system (Fig. 3A,B): This tissue is the unit of *Drosophila* oogenesis that with time produces oocytes of a several hundred micron scale, rendering this model challenging for microscopy. On the other hand, a single egg chamber is composed of two tissue types: germline (oocyte and the nurse cells) and soma (the follicular epithelium that surrounds the germline) (Fig. 3A). As these cells differ in origin and function, and each has a plethora of cell-type specific transcripts, they constitute ideal internal negative controls for each other in the smFISH assay.

We first compared the raw signal of a set of 42 different, enzymatically labeled probes targeting the germline specific *oskar* mRNA (osk42×-633, Fig. 3A) to that of a set of 45 chemically labeled, conventional smFISH probes (osk3P(45×)-633, Fig. 3B) previously used in smFISH assays (Little et al. 2015). We found that our Atto633 labeled probe set—although it contained only 42 different oligonucleotide species—provided a slightly brighter signal than the reference osk3P(45×)–Atto633 set (Fig. 3A,B). This unexpected difference was likely due to the fact that our enzymatically labeled probes were freshly produced, whereas the reference osk3P set was already several years old at the time of the experiment. Furthermore, we observed similar low aspecific backgrounds in the *oskar* nonexpressing follicle cells with both probe sets (Fig. 3A,B).

To test the single-molecule sensitivity of our probe sets, we labeled *oskar* mRNA with two different fluorophores coupled to two probe sets (osk18×–Atto633 and osk17×–Atto565, the probes are arranged in an alternating setup, Supplemental Table S1). As shown in Figure 3C, there was a high degree of overlap between the two colors in the germline, but hardly any puncta were observed in the follicle cell layer with either of the fluorophores. Similarly, another germline specific mRNA, *nanos*, was detected almost exclusively in the nurse cells; however, no apparent colocalization was observed between the nos18×–Atto633 and osk17×–Atto565, consistent with previous observations (Little et al. 2015) indicating specificity of these probe sets (Fig. 3D).

We subsequently analyzed automatically detected smFISH objects using one of the colors as the reference, the other as the target channel. By comparing the object densities, we found 20- to 100-fold more objects in the transcript-expressing germline relative to the nonexpressing follicle cell layer (Fig. 3E; Supplemental Fig. S3B), indicating that the false positive detection rate (FPDR) of these probe sets is ∼5%–1%. Next, we assayed the single-molecule codetection rate of the two differently labeled probe sets. Since in the nurse cells *oskar* mRNA was shown to be present in RNPs containing single and two copies of the mRNA (Little et al. 2015), we first fitted the signal intensity distribution of the reference channel with Gaussian functions as described in Supplemental Figure S2 and Little et al. (2015). We considered that objects with a fluorescence intensity lower than the µ + 2σ of the first fitted Gaussian function contained a single mRNA molecule (Fig. 3D,E; Supplemental Fig. S2, dashed vertical lines). Within this population of objects, we estimated a detection threshold of the target channel (see legend of Fig. 3F,G). We found that ∼91% of objects detected by osk18×–Atto633 would be codetected by osk17×–Atto565; ∼86% codetection rate in the complementary analysis (Supplemental Fig. S3C). These values are in good agreement with what has been previously observed using chemically synthesized smFISH probes (Raj et al. 2008; Oka and Sato 2015; Stapel et al. 2016). On the other hand, only 27% of nos18×–Atto633 positive objects showed detectable osk17×–Atto565 fluorescence, and there was no linear relation between the signal intensities of these two probes (*R*^2^ = 0.006).

To assess the performance of our gfp probe set, we performed similar codetection analysis on egg-chambers that expressed *oskar-EGFP* mRNA in addition to the endogenous *oskar* mRNA (Supplemental Fig. S3A). Despite the relatively high FPDR of our gfp23×–Atto633 probe set (∼10%, Supplemental Fig. S3B)—probably due to the considerably higher *T*_m_ of the gfp probes than the osk probes—we found that ∼78% of gfp23×–Atto633 positive objects were codetected by osk42×–Atto565 (Supplemental Fig. S3D).

**FIGURE 4.**
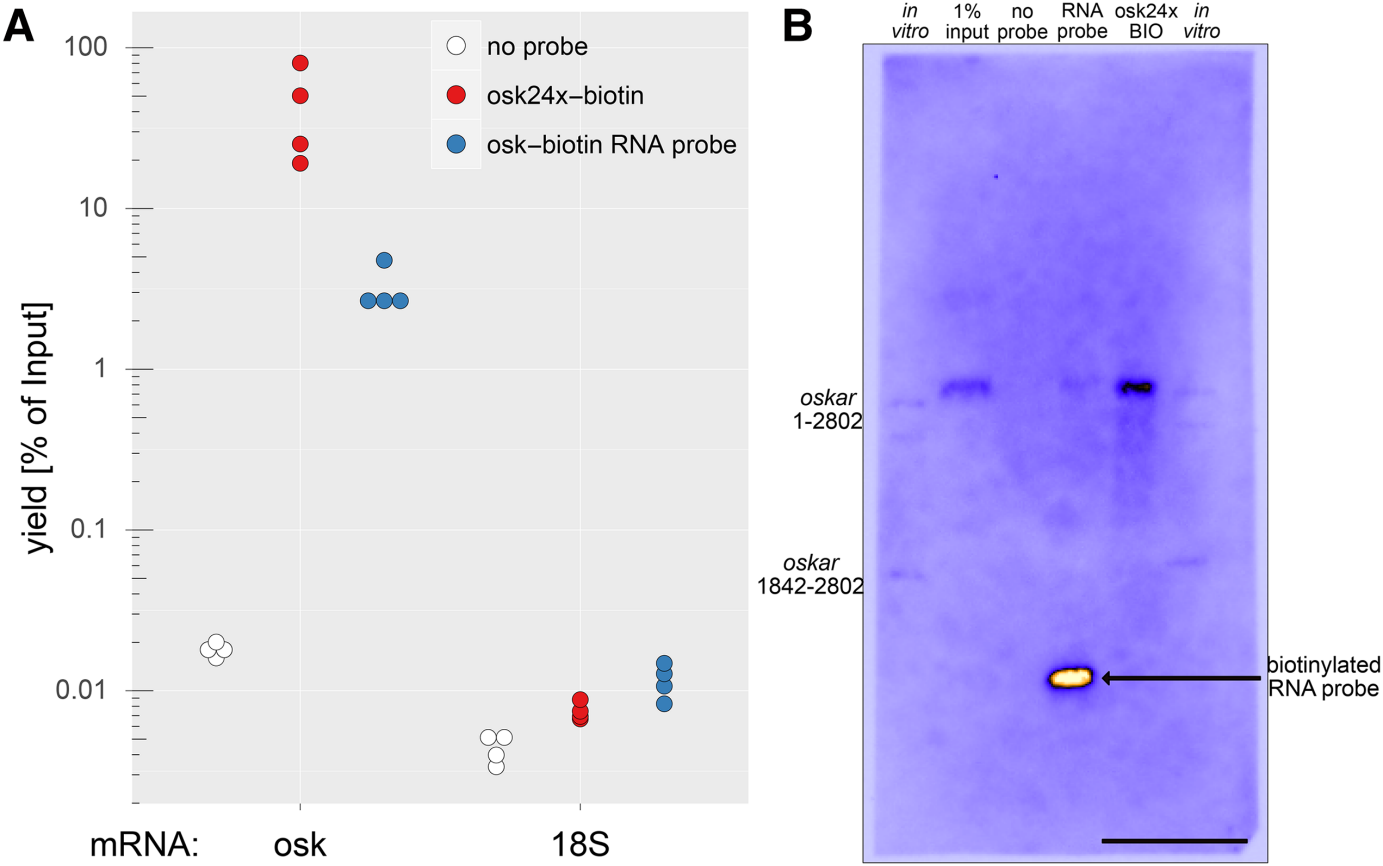
mRNA affinity purification using biotinylated DNA versus RNA probes. (*A*) Quantitative RT-PCR analysis of the captured RNA. A biotinylated DNA probe set (osk24×–biotin) captured *oskar* mRNA more efficiently than a single, 400-nt-long, internally biotinylated RNA probe (osk–biotin RNA probe), whereas the unrelated RNA (18S ribosomal RNA) was only marginally captured. (*B*) Northern blot of the captured *oskar* mRNA (4 min exposure). Equal volumes of the 1% input (lane *2*, 12.5 µg total RNA) and 20%–20% of the three eluates (lanes *3*–*5*) were blotted to a membrane, and *oskar* mRNA was hybridized with the osk59×–biotin probe set. The signal was detected by HRP-conjugated streptavidin that recognized the biotinylated 400-nt-long RNA probe used for the RNA capture in lane *4*. As markers, in vitro transcribed *oskar*^*1-2802*^ (almost full-length RNA, 150 pg/lane) and *oskar*^*1842-2802*^ (almost the entire *oskar* 3′ UTR, 300 pg/lane) was used (lanes *1*,*6*). Note that *oskar*^*1-2802*^ ran as a doublet, possibly because of two stable RNA conformations. Scale bar is 1 cm.

Biotinylated DNA probes have been used previously to isolate RNA from complex samples by in solution hybridization (Yehle et al. 1987; Chu et al. 2015; McHugh et al. 2015; Minajigi et al. 2015). Therefore, we wanted to assess the performance of our biotinylated DNA probe set (osk24×–biotin) to purify endogenous *oskar* mRNA from *Drosophila* ovarian lysates. Compared with a single 400-nt-long in vitro synthesized antisense RNA probe that has incorporated multiple biotinylated UTPs (approximately 11 per RNA) at various positions, equimolar amounts of osk24×–biotin probes were capable of capturing approximately 10-fold more endogenous *oskar* mRNA as determined by qRT-PCR (Fig. 4A). Furthermore, we found that osk24×–biotin probes selectively captured the desired mRNA with very little cross-contamination, as judged by the amounts of copurified 18S ribosomal RNA.

To assay if there was a considerable amount of RNA degradation during the RNA capture—which could be expected especially when RNA/DNA hybrids are formed—we performed Northern-blot analysis of the eluted RNA using the full set of biotinylated *oskar* probes (osk59×–biotin) to detect *oskar* mRNA. As shown in Figure 4B, we found that although there is a detectable smear of the signal when the RNA was captured by the osk24×-biotin probe set, the vast majority of captured *oskar* mRNA appeared to be intact. As our detection method could identify small amounts of short pieces of *oskar* mRNA (i.e., 300 pg *oskar*^*1842-2802*^ targeted by only 15 different probes), we conclude that RNA degradation is not a major issue in the RNA capture assay.

In summary, self-made biotinylated DNA probes are cost-efficient tools that can be used for biochemical purification and subsequent analysis of endogenous mRNAs.

## DISCUSSION

The method we have presented here enables conversion of an array of inexpensive PCR oligos complementary to a given RNA target into functional smFISH or RAP probes adapted to the experimenter's specific needs. Although we focused on the optimization of Atto565 and Atto633 labeling, as shown in Figure 1 and Supplemental Figure S1, quite a number of the different dyes we tested are suitable for use with this method. Also, as exemplified by the different combinations of *oskar* probes we used in this and a previous (Gáspár et al. 2017) study, the method offers unprecedented flexibility in choosing and manipulating the composition of the generated probe sets. Importantly, as opposed to labeling primary amine containing oligonucleotides, here we convert the volatile dye–NHS ester into a stable dye/biotin–ddUTP conjugate that can be thawed/frozen multiple times and stored without perceptible loss of activity (our oldest aliquot in use is an over 1-yr-old Atto633–ddUTP conjugate).

Another important aspect of this method is that the associated specific cost of a labeling reaction is low: A typical 1000-pmol scale synthesis of a set of 30 oligos usually yields probes sufficient for ∼100 smFISH experiments (using 2.5 nM/probe final concentration in a 100 µL hybridization volume). The costs of the initial set of 30 ssDNA oligos (typical delivery amount is ∼30 × 20,000 pmol) is usually a two-digit figure (in USD). The expensive components of the labeling reaction are sufficient for ∼40–80 (TdT, 500 U), ∼100 (1 µmol Atto565–NHS ester), and ∼200 (1 µmol ddUTP) of such 1000 pmol scale reactions, and thus cost below 10 USD including production and purification.

Taken together, as the entire synthesis and purification takes ∼24 h and requires no special equipment or prior expertise, we consider this method a good alternative to commercially available smFISH probe sets.

Recently, other cost-reducing RNA detection techniques with single-molecule sensitivity have been developed, such as the single-molecule inexpensive FISH (smiFISH) (Tsanov et al. 2016) and single-molecule hybridization chain reaction (smHCR) (Shah et al. 2016). These two techniques also use inexpensive unlabeled oligonucleotides to initiate the RNA detection. The initiator molecules are then detected by a secondary round of hybridization with a single (smiFISH) or a pair of labeled probe species (smHCR) establishing RNA detection systems that are similarly flexible (e.g., change of colors) to our probe labeling setup. smiFISH uses a set of primary probes composed of a target specific 5′ and a system heterologous 3′ sequence shared among the probes. This shared sequence is detected by a common, doubly labeled secondary probe per target RNA. Therefore, smiFISH produces a signal roughly twice as bright as smFISH carried out by the same number of singly labeled probes, with an identical signal-to-noise ratio. smiFISH also preserves the quantitative feature of smFISH, which directly relates signal intensity to RNA copy number (Tsanov et al. 2016). smHCR, on the other hand, has greatly increased brightness due to the series of fluorophores deployed around the target during the chain reaction. Although this allows easier detection of the signal—particularly when detecting RNA in a highly light-scattering environment—it also increases the likelihood of false positive detection, and it is not yet clear whether the signal intensity produced by smHCR can be directly related to RNA copy number (Shah et al. 2016). Finally, both of these methods require considerably longer (55–60 nt) oligos—typically 24–48 per target—whose cost (production, purification) is on par with that of 96–100 oligos used in our probe labeling method. Nevertheless, these cost-efficient methods—smiFISH, smHCR, and ours, which allows easy and cheap production of labeled oligonucleotides for quantitative RNA detection—will find their appropriate niches in the toolbox of every RNA biologist.

## MATERIALS AND METHODS

### Synthesis of labeled ddUTP

Amino-11-ddUTP (NH_2_-ddUTP, Lumiprobe, stock concentration 10 mM) was combined with twofold molar excess of dye–NHS-ester in the presence of 0.1 M NaHCO_3_, pH 8.3, and the reaction mixture was incubated for 2–3 h at room temperature isolated from light. Subsequently, 1 M Tris HCl, pH 7.4 was added to 10 mM final concentration in order to quench any unreacted NHS-ester groups. The reaction mixture was adjusted with nuclease-free H_2_O to obtain 2–5 mM final concentration of the labeled-ddUTP. The following dye–NHS esters were used: Atto425–NHS, Atto465–NHS, Atto488–NHS, Atto565–NHS, Atto633–NHS, AttoRho14–NHS, and Atto725–NHS were purchased from Atto-tec GmbH; Abberior 470SX-NHS, Abberior RED-NHS, and Abberior 635P-NHS esters were a gift of Abberior GmbH; BDP-FL-NHS-ester was obtained from Lumiprobe. The NHS esters were reconstituted with anhydrous DMSO to 20–40 mM concentration in a chamber filled with Blue Silica desiccant to prevent moisture contamination of the NHS-ester. AlexaFluor488–NHS-ester was obtained from Life Technologies, Inc. and was reconstituted to 10 mM concentration. Biotin–NHS-ester was obtained from Sigma-Aldrich GmbH and reconstituted to 20–40 mM final concentration. Typically, the totality of the reconstituted NHS-ester was immediately put in reaction with NH_2_-ddUTP. In the initial experiments, several months old aliquots of Atto488–NHS and Atto633–NHS esters were used, reconstituted to 2 mM. Depending on the initial concentration of the reconstituted dye, the final DMSO content of the labeled-ddUTP was between 25% and 80%.

### Enzymatic production of labeled oligonucleotides

Nonoverlapping arrays of 18–22mer DNA oligos complementary to *oskar*, *nanos*, *gfp*, and 18S RNA targets were manually selected (Supplemental Table S1). The selection criteria included 45%–60% GC content, similar melting temperature, and the oligos were designed such that in most cases the 3′ incorporated ddU was also part of the hybrid. Adjacent oligos were separated by at least 2 nt. Desalted or reverse phase cartridge purified PCR oligos were obtained from Sigma-Aldrich GmbH and reconstituted to 240–250 µM with nuclease-free H_2_O. Of note, 500–3000 pmol of individual or pooled oligonucleotides were mixed with 1.5- to fivefold molar excess of the labeled ddUTPs (as indicated in the Results and in the figure legends) in 1× TdT buffer. The mixture was completed by the addition of TdT enzyme (0.006 U/pmol ssDNA, standard amount) and was incubated at 37°C typically overnight in a PCR thermocycler with hot lid on. The provided Excel sheet (Supplemental Table S2) provides the precise composition of the reaction mixture for the optimized Atto565, Atto633, and biotin labeling.

### Purification of labeled oligonucleotides

Upon completion of the enzymatic reaction, the reaction mixtures were supplemented with sodium acetate, pH 5.5 (300 mM final concentration), 1.5–5.0 µg linear acrylamide (Thermo Fisher Scientific) depending on the synthesis scale (e.g., 2.5 µg for a 1000 pmol, or 5 µg for a 3000 pmol scale synthesis), and diluted to 200 µL with nuclease-free H_2_O. This mix was transferred into a conventional 1.5 mL Eppendorf tube and 800 µL −20°C 100% ethanol was added and mixed well. Precipitation was facilitated by incubating the mixture at −80°C for 15–20 min. Precipitated oligos were pelleted by centrifuging with 16000*g* at 4°C for 20 min. The pellet was washed in 1 mL 4°C 80% ethanol by vortexing and pelleting with 16,000*g* at 4°C for 20 min. After the first wash, the pellet was transferred into a clean 1.5 mL Eppendorf tube and the washing was repeated two more times. Subsequently, residual traces of ethanol were removed and the pellet was air-dried before reconstitution with nuclease-free H_2_O.

### Analysis of the labeled oligonucleotides

Degree-of-labeling (DOL), concentration (*c*), and recovery efficiency (recovery%) of the labeled oligonucleotides were assessed by UV–Vis spectroscopy using a NanoDrop spectrophotometer. Absorbance at 260 nm (OD_260_) and at the dye absorption maximum (OD_dye_) were measured and the following formulae were used to estimate properties of the labeled oligonucleotide:

(1) $$c=\frac{\text{O}{\text{D}}_{260}-cf_{260}\times \text{O}{\text{D}}_{\mathrm{dye}}}{\varepsilon_{\mathrm{oligo}}},$$

(2) $$\text{DOL}=\frac{\text{O}{\text{D}}_{\mathrm{dye}}}{\varepsilon_{\mathrm{dye}}}\times c^{-1},$$

(3) $$\text{Recovery}\%=\frac{c\times V}{n_{\mathrm{initial}}}.$$

ε_dye_ and *cf*_260_ are the molar extinction coefficient and the relative absorbance at 260 nm of the dye, respectively. ε_oligo_ is calculated by taking the average of the extinction coefficient of oligonucleotides in the oligo mixture and adding 9000 mol/cm corresponding to the incorporated ddU. *V* is the reconstituted volume of the labeled oligo; *n*_initial_ is the starting amount of the unlabeled oligo.

To determine the degree of incorporation of biotin, we used the HABA/Avidin assay (Sigma-Aldrich) that is based on the displacement of HABA from avidin by biotin, resulting in a loss of color that can be quantified by measuring the absorbance at 500 nm. In the case of all our biotinylated probe sets, we measured close to equimolar amounts of biotin and oligonucleotides present in the samples (DOL ∼ 1).

Denaturing PAGE analysis was performed on labeled single oligonucleotides or pooled oligonucleotides of the same length. Of note, 15–96 pmol of labeled product were denaturated by boiling in the presence of 4 M urea and 1× DNA loading dye for 10 min and were immediately loaded to a 15% polyacrylamide gel containing 1× TBE and 8 M Urea. The gel was prerun for at least 30 min at 30 V/cm and, once loaded, the same voltage was used to separate the denatured oligonucleotides using the unlabeled oligo as the size marker. Gels were imaged with a BioRad ChemiDoc gel documentation system using 254 nm excitation and a long-pass visible light filter to detect dye fluorescence. Subsequently, gels were stained with 2× SYBR GOLD in 1× TBE for 10 min and imaged with a Peqlab gel documentation system using 254 nm excitation and a band-pass visible light filter. This allowed visualization of the unlabeled fraction of the oligo in addition to the labeled product. Due to the band-pass filter used and the possibly highly efficient energy transfer between SYBR GOLD and the far red dyes, the Atto633 and Abberior RED labeled products are not visible in the stained gels (Fig. 1F′; Supplemental Fig. S1). The two images (before and after staining) were aligned in ImageJ using the gel boundaries as transformation landmarks. To determine DOL by PAGE, the amount of unlabeled oligo (*n*_unlabeled_) was determined by comparing the fluorescence of the corresponding band to a series of unlabeled oligo of known quantity (typically 3, 6, and 9 pmol). DOL was calculated as follows: *1* − *n*_unlabeled_/*n*_loaded_.

### smFISH and image analysis

The following probe sets were used during smFISH: osk17×–Atto565 (DOL = 0.93), osk18×–Atto633 (DOL = 1.07), nos18×–Atto633 (DOL = 1.07), osk42×–Atto565 (DOL = 0.96), gfp23×–Atto633 (DOL = 0.94). Single-molecule FISH was performed similarly as described in Gáspár et al. (2017) using ovaries of *w*^*1118*^ and *oskar-EGFP* expressing (Sarov et al. 2016) female flies. Briefly, ovaries were dissected into 2 v/v% PFA, 0.05 v/v% Triton X-100 in PBS (pH 7.4) and were fixed for 20 min on an orbital shaker. The fixative was removed and the ovaries were washed twice in PBT (PBS + 0.1 v/v% Triton X-100, pH 7.4) for 5 min. *w*^*1118*^ samples were treated with 2 µg/mL proteinase K in PBT for 5 min and then were subjected to 95°C in PBS + 0.05 v/v% SDS for 5 min. Specimens were cooled by adding 2× volume of room temperature PBT. Proteinase K/heat treatment was omitted in the case of *oskar-EGFP* expressing samples so as to preserve GFP fluorescence. Ovaries were prehybridized in 200 µL 2×HYBEC (300 mM NaCl, 30 mM sodium citrate pH 7.0, 15 v/v% ethylene carbonate, 1 mM EDTA, 50 µg/mL heparin, 100 µg/mL salmon sperm DNA, 1 v/v% Triton X-100) for 10 min at 42°C. Fifty microliters of prewarmed probe mixture (12.5–25 nM per individual oligonucleotide) was added to the prehybridization mixture, and hybridization was allowed to proceed for 2 h at 42°C. Free probe molecules were washed out of the specimen by a series of washes: 0.5 mL prewarmed 2×HYBEC, 1 mL prewarmed 2×HYBEC:PBT 1:1 mixture, 1 mL prewarmed PBT for 10 min at 42°C, and finally 1 mL prewarmed PBT allowed to cool down to room temperature. Ovaries were mounted in 80 v/v% 2,2-thiodiethanol in PBS.

Stacks of images were acquired on a Leica TCS SP8 confocal microscope using a 63× 1.4 NA oil immersion objective and were restored by deconvolution in Huygens Essential. Deconvolved images were analyzed in ImageJ using a custom-made particle detection and tracking algorithm (Gaspar et al. 2014, 2017). Briefly, the algorithm finds in each slice of the reference channel the local maxima that represent the upper few percentiles of the signal distribution. These 2D objects are then connected along the *z*-axis based on their center-positions to create 3D objects. Signal intensities of both the reference and target channels of all 3D objects with a minimum of three slices depth (smFISH object) found within the nurse cell compartment were recorded and were subject to statistical analyses in R (as described in the legend of Fig. 3). To determine FPDR, smFISH object density in manually selected regions in the follicle cells was compared with the smFISH object density in the nurse cells in randomly selected regions of comparable volumes.

### RNA capture method

Ovaries from well-fed *Drosophila melanogaster* (Oregon R) were resuspended in 3× volume of lysis buffer (50 mM Tris–HCl pH 7.0, 10 mM EDTA, 1 v/v% SDS, supplemented with fresh PMSF 1 mM, cOmplete mini EDTA-free protease inhibitor [Roche] and RiboLock RNase Inhibitor [Thermo]) and mechanically homogenized. The lysates were cleared by centrifugation (5 min at 140*g*). The supernatant was further diluted with 2 volumes (2:1) hybridization buffer (750 mM NaCl, 1 v/v% SDS, 50 mM Tris–HCl, pH 7.0, 1 mM EDTA, 15 v/v% ethylene carbonate [Sigma-Aldrich], fresh 1 mM PMSF, cOmplete mini EDTA-free protease inhibitor [Roche], and RiboLock RNase Inhibitor [Thermo]), precleared with Pierce Avidin Agarose (Thermo) for 30 min and cleared by centrifugation (5 min at 140*g*). The precleared lysates were supplemented with 0.25 µg of biotinylated probes (osk24×-biotin) or of a 400-nt-long biotinylated RNA complementary to the 3′ of the *oskar* cds (Gaspar et al. 2014) per milliliter of ovaries and incubated at 37°C for 2 h with constant rotating. RNA–probe complexes were collected by the addition of magnetic Streptavidin beads (Dynabeads MyOne C1, Thermo) for 1 h at 37°C. The beads were washed three times for 5 min at 37°C with low salt wash buffer (300 mM NaCl, 30 mM sodium citrate pH 7.0, 0.5 v/v % SDS, fresh PMSF, and cOmplete mini EDTA-free protease inhibitor [Roche]), 2× high salt wash buffer (750 mM NaCl, 30 mM sodium citrate pH 7.0, 0.5 v/v% SDS, fresh PMSF, and cOmplete mini EDTA-free protease inhibitor [Roche]) and 2× low salt wash buffer. The RNA was eluted in TE buffer (10 mM Tris–HCl, pH 7.0, 1 mM EDTA) at 95°C for 5 min and extracted with the Quick-RNA MicroPrep Kit (Zymo Research). cDNA was synthesized using SuperScript III First-Strand Synthesis SuperMix (Thermo) with random hexamer primers. Real-time PCR analysis was carried out using SYRB Green PCR Master Mix (Applied Biosystems) on a StepOnePlus Real-Time PCR System (Applied Biosystems) with primers against *oskar* (forward: 5′-CAGACTCTTCTCGTCCACTCAG-3′ and reverse: 5′-CGTGCAGTGGAAATGGATTGC-3′ flanking the first, 248-nt-long intron; the amplicon is outside of the probe-targeted portion of the RNA) or 18S rRNA (forward: 5′-CGGAGAGGGAGCCTGAGAA-3′, reverse: 5′-AGCTGGGAGTGGGTAATTTACG-3′). The results were compared with a dilution series of input samples to calculate the yield of the capturing procedure.

### Northern blot analysis

Eluted RNA was mixed with Gel Loading Buffer II (Ambion) in a one-to-one ratio, then loaded on a 1% native agarose gel in 0.5× TBE. Electrophoresis was carried out for 45 min at 76 V/∼18 mA in 0.5× TBE. The RNA was blotted onto a HyBond-N+ nitrocellulose membrane (GE Healthcare) in 0.5× TBE in a wet-transfer tank (BioRad) (45 min 200 mA/∼40 V). The RNA was cross-linked to the membrane by 40,000 µJ/cm^2^ UV_254nm_ (Startagene UV Stratalinker 2400). The membrane was prehybridized in Northern HYB buffer (300 mM NaCl, 30 mM sodium citrate pH 7.0, 15 v/v% ethylene carbonate, 1 mM EDTA, 50 µg/mL heparin, 17 nM/probe unlabeled gfp19nt + gfp20nt, 0.5 v/v% SDS) for 15 min at 37°C with continuous rocking. Of note, 1 nM/probe final concentration of osk59×–biotin in Northern HYB buffer prewarmed to 37°C was applied for 75 min, and the free probe was removed by washing in prewarmed Northern HYB buffer (10 min), followed by 2× SSC (10 min). The membrane was rinsed in IBEX (10 mM HEPES, pH = 7.7, 120 mM KCl, 1 mM EDTA, 0.3% Triton X-100) at RT and was blocked for 15 min in 1× Western-blocking reagent (Roche) in IBEX. HRP-conjugated streptavidin (PerkinElmer) diluted 1600-fold in 1× Western-blocking reagent in IBEX was applied for 60 min and was removed in 4 × 10 min washes in IBEX and a final rinse in PBS + 0.1% Tween-20. The signal was developed using Western Lightning Plus ECL reagent (PerkinElmer) and was recorded with the BioRad ChemiDoc gel documentation system.

### In vitro transcription

Templates of *oskar*^*1-2802*^ and *oskar*^*1842-2802*^ were amplified from a full-length *oskar* cDNA cloned into a pBluescript vector using 5′-TGAATTGTAATACGACTCACTATAGGGAGAGGATCACTTTCCTCCAAGCG-3′ and 5′-TGAATTGTAATACGACTCACTATAGGGAGAGTTGGGTTCTTAATCAAGATAC-3′ forward primers (T7 promoter is underlined), respectively, and a common 5′-AACGTGATCACCATCAATAC-3′ reverse primer. In vitro T7 transcription and RNA purification was carried out using the NEB HiScribe T7 High Yield Transcription Kit according to the manufacturer's instructions.

### Statistical analyses

All statistical analyses (indicated in the Results and in the figure legends) were carried out in R (R Core Team 2012) using RStudio (https://www.rstudio.com). A multiple Gaussian model was fitted to the smFISH signal intensity distributions using the mixtools package (https://cran.r-project.org/web/packages/mixtools/index.html). All graphs were plotted by the ggplot2 library in R (Wickham 2009).

## SUPPLEMENTAL MATERIAL

Supplemental material is available for this article.

## Supplementary Material

Supplemental Material
