# Supplementary material for: Enzymatic production of single-molecule FISH and RNA capture probes
Source: RNA. 2017 Oct;23(10):1582–91. doi: 10.1261/rna.061184.117 (PMC5602115; doi:10.1261/rna.061184.117)
Supplement: Supplemental Material [file supp_23_10_1582__index.html]

Enzymatic production of single-molecule FISH and RNA capture probes — Supplemental Material 

# Enzymatic production of single-molecule FISH and RNA capture probes

## Supplemental Material

- Supplemental\_Figures.pdf
- Supp\_TableS2.xlsx
