# Supplementary material for: Enzymatic production of single-molecule FISH and RNA capture probes
Source: RNA. 2017 Oct;23(10):1582–91. doi: 10.1261/rna.061184.117 (PMC5602115; doi:10.1261/rna.061184.117)
Supplement: Supplemental Material [file supp_061184.117_Supplemental_Figures.pdf]

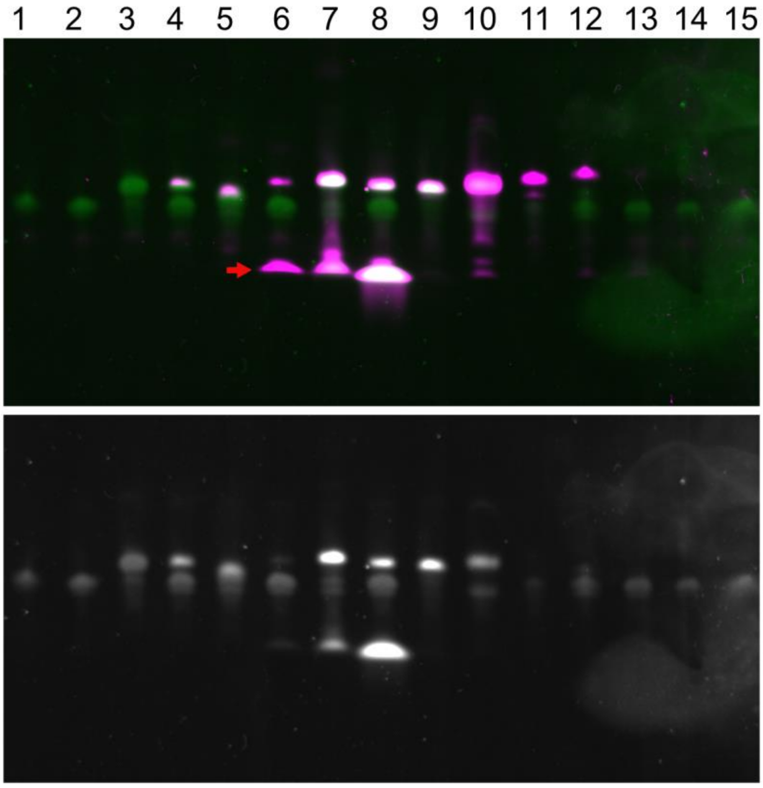

Figure S1: 3′ incorporation of labelled ddUTP by TdT.

Labelling of osk20nt-15x probe mixture (1, 2, 14, 15) using biotin-ddUTP (3), Atto425-ddUTP (4), Atto465-ddUTP (5), Abberior 470SX-ddUTP (6), Atto488-ddUTP (7), AlexaFluor488-ddUTP (8), BDP-FL-ddUTP (9), Atto565-ddUTP(110), Atto633-ddUTP (11), Abberior RED-ddUTP (12) and Abberior 635P-ddUTP (13). Labelled ddUTP and TdT were used in 4-fold molar excess and in standard amounts, respectively. Red arrow indicates free dye-ddUTP carry-over. Lanes were loaded with 3 pmol (1, 14), 6 pmol (2, 15) and 15 pmol oligo/lane (3-13), respectively.

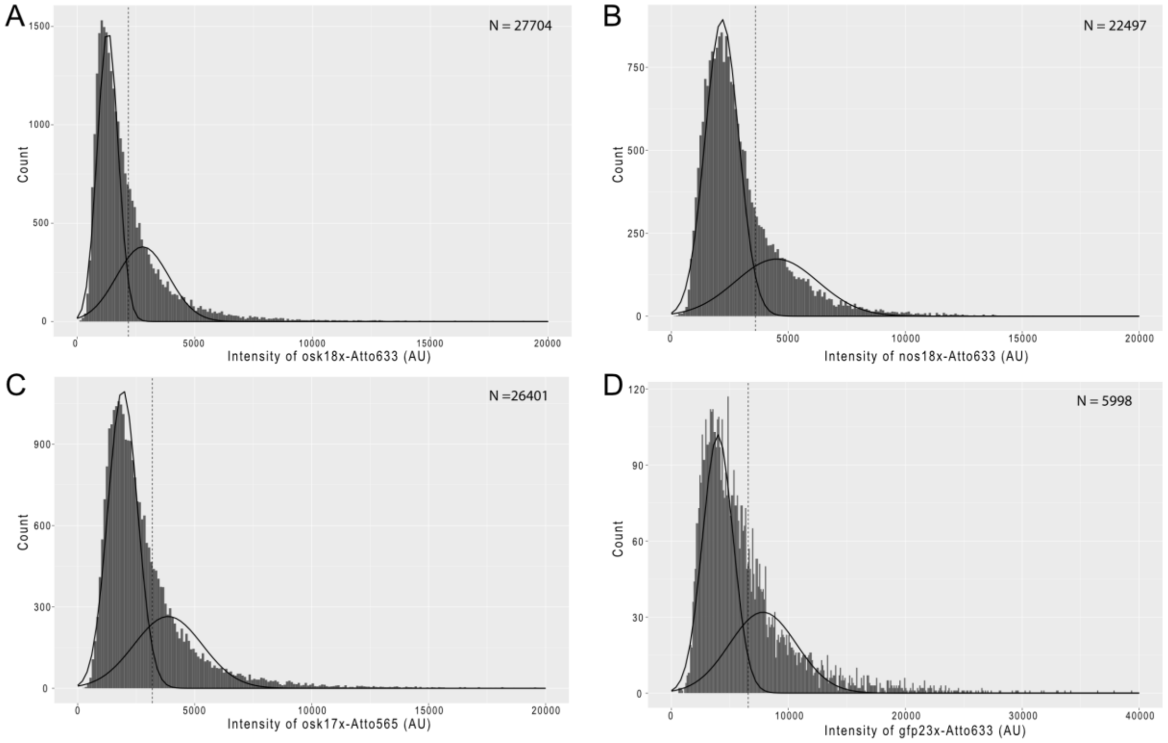

Figure S2: Intensity distribution of the smFISH signals

A-D - Intensity distribution of the smFISH signals of the osk18x-Atto633 (A), nos18x-Atto633 (B), osk17x-Atto565 (C) and gfp23x-Atto633 (D) probe sets. The distribution histograms were fitted by Gaussian functions and the two functions with the smallest μ-values are proportionally represented (solid curves) (5). The difference between these smallest μ-values is ~ 2-fold in all four cases. The sum of smallest μ and the double of the corresponding σ-value ($\mu_1+2\sigma_1$) was taken as the intensity threshold of single mRNA containing smFISH objects (dashed vertical lines). Numbers indicate the number of objects analyzed per experiment.

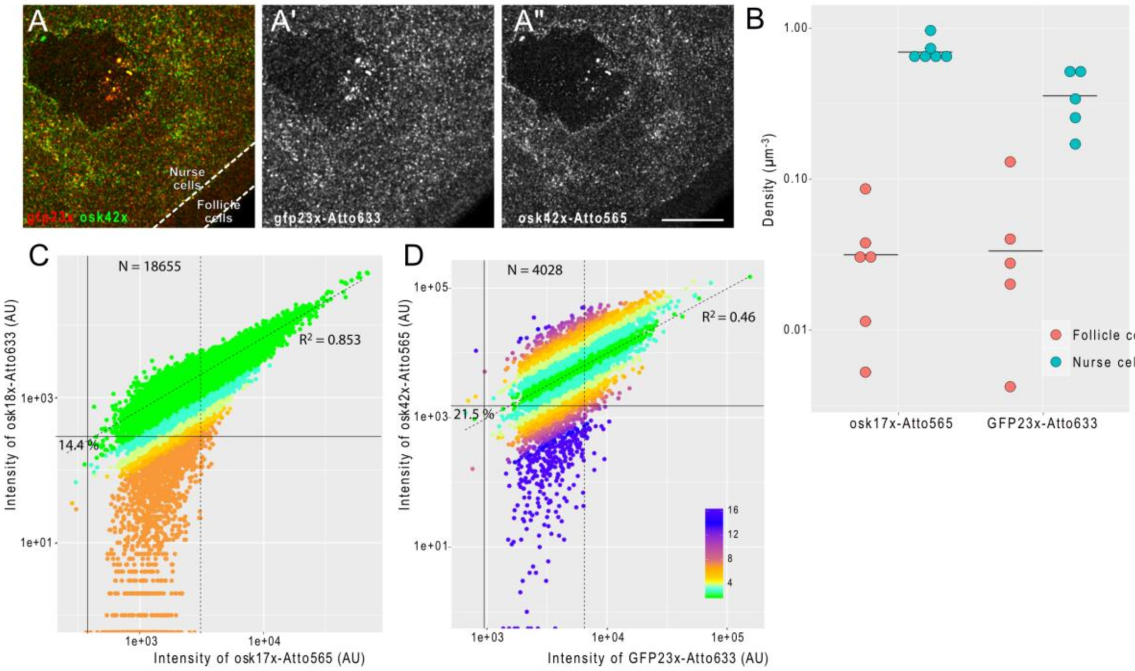

Figure S3. smFISH using 3′ end labelled probe mixtures

A-A” – smFISH of *gfp* and *oskar* mRNA in a developing *Drosophila* egg-chamber that expresses both endogenous *oskar* and transgenic *oskar-EGFP* mRNAs using gfp23x-Atto633 (red, A’) and osk42x-Atto565 (green, A”) probe sets. The two probes sets label different portions of *oskar-EGFP* mRNA, while endogenous *oskar* mRNA is labelled exclusively by the osk42x-Atto565 probe set. The germline expressing *oskar(-EGFP)* mRNAs (nurse cells) and the soma (follicle cells) are indicated. Scale bar is 10 µm.

B – Density of detected smFISH signal in the mRNA expressing (cyan, nurse cells) and non-expressing (red, follicle cells) compartments of the egg-chambers. Horizontal lines indicate the mean value of observations.

C, D – Intensity of osk18x-Atto633 (C) and osk42x-Atto565 (D) probe sets (target channel) as a function of signal intensity of smFISH objects detected by the fluorescence of osk17x-Atto565 (C) or gfp23x-Atto633 (D) probe sets (reference channel). Percentage values represent the fraction of single mRNA containing smFISH objects whose target channel signal intensity falls below the estimated detection threshold (solid horizontal line, described in the legend of Figure 3D and E). Numbers of these single copy mRNA objects are indicated in the graphs. Colors represent the relative difference of the observed and expected target channel signals (see panel D for key).

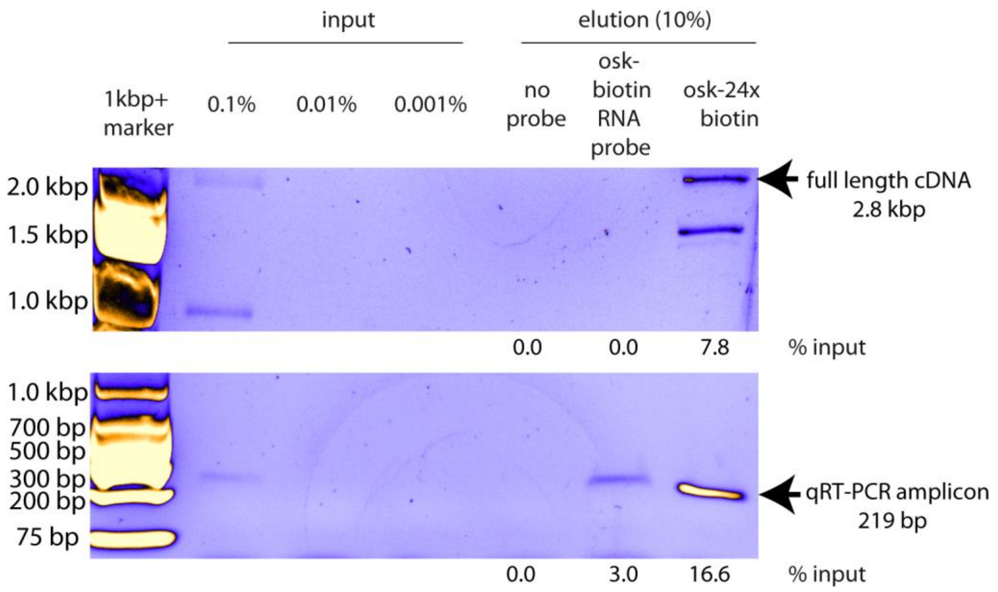

Figure S4. Semi-quantitative RT-PCR of *oskar* cDNA. An almost full-length cDNA (upper panel) and the qRT_PCR amplicon (lower panel) were amplified from the reverse transcribed samples (as indicated above the lanes) using 36 and 16 cycles, respectively. The amount of the specific product with the appropriate size relative to that of the input was determined by densitometry of the scanned signal. Note that the qRT-PCR amplicon suggests no or very small amount of genomic DNA/pre-mRNA contamination indicated by the absence of a 457 bp long product containing the first intron of *oskar* mRNA. We did not obtain any full-length cDNA from the RNA probe eluate, possibly because the 400 nucleotide long RNA/RNA hybrid prevented the full length reverse transcription. Marker (Thermo GeneRuler 1kbp+) is indicated on the left.

Table S1: List of the oligonucleotides and pools of oligonucleotides used in this study.

**Name**	**Sequence : (5' to 3')**	**Length**	**nos18x**					
nos3'UTR#1	TGCTGGCGGTTGTTTCATG	19	+					
nos3'UTR#2	GTAGCCGACGACGAAAGTG	19	+					
nos3'UTR#3	TTTACGAAATGAAGGCGACCAG	22	+					
nos3'UTR#4	GCCACGACGATTGAACAAG	19	+					
nos3'UTR#5	CTTGGATTTGAGTGATCGTTCG	22	+					
nos3'UTR#6	TAGGCACGGGATAACGCTC	19	+					
nos5'UTR#1	AAGTTACGGTTATCGCGCAC	20	+					
nos5'UTR#2	GGTAAAGCTACGCGCCAAC	19	+					
nosCD#1	CTTAATCGTGTGCGCCGAG	19	+					
nosCD#2	CTCGCACTGAGTGGCTATTG	20	+					
nosCD#3	CTGATCTCTTTGGCCTTGCTG	21	+					
nosCD#4	CCGAGATTGGTGGACACAG	19	+					
nosCD#5	CCACACGTTGTTCAGATGC	19	+					
nosCD#6	TTAAGTTGCCGCCATTGGTC	20	+					
nosCD#7	CAATCTCGTCCGTTTGCTGG	20	+					
nosCD#8	CTGGAGCAGCAAGTGGTAG	19	+					
nosCD#9	CGTAATGGGCGGACTCAAAG	20	+					
nosCD#10	CCAGAATGTTGAGCCCTCC	19	+					
			**gfp-20nt-7x**	**gfp-19nt-11x**	**gfp23x**			
EGFP#1	TCCTCGCCCTTGCTCACCA	19		+	+			
EGFP#2	GTTTACGTCGCCGTCCAGC	19		+	+			
EGFP#3	CCGGACACGCTGAACTTG	18						
EGFP#4	CAGCTTGCCGTAGGTGGCA	19		+	+			
EGFP#5	GCCGGTGGTGCAGATGAAC	19		+	+			
EGFP#6	AAGCACTGCACGCCGTAGG	19		+	+			
EGFP#7	CTGCTTCATGTGGTCGGGG	19		+	+			

EGFP#8	TGGACGTAGCCTTCGGGCA	19		+	+			
EGFP#9	GCCGTCGTCCTTGAAGAAGA	20	+		+			
EGFP#10	GTCGCCCTCGAACTTCACC	19		+	+			
EGFP#11	TCGATGCCCTTCAGCTCGA	19		+	+			
EGFP#12	CAGGATGTTGCCGTCCTCC	19		+	+			
EGFP#13	AGACGTTGTGGCTGTTGTAG	20	+		+			
EGFP#14	GTTCTTCTGCTTGTCGGCCA	20	+		+			
EGFP#15	GGCGGATCTTGAAGTTCACC	20	+		+			
EGFP#16	GGGTGTTCTGCTGGTAGTGG	20	+		+			
EGFP#17	GGGTGCTCAGGTAGTGGTTG	20	+		+			
EGFP#18	GCTTCTCGTTGGGGTCTTTGC	21						
EGFP#19	CGAACTCCAGCAGGACCATG	20	+		+			
EGFP#20	TCGTCCATGCCGAGAGTGA	19		+	+			
EGFP#21	TCTCGTTGGGGTCTTTGC	18			+			
EGFP#22	CGCTGCCGTCCTCGATGT	18			+			
EGFP#23	GGGTGGTCACGAGGGTGG	18			+			
*EGFP#24**	*AAGTTCAGCGTGTCCGGC*	*18*			+			
*EGFP#25**	*GTGGTGCCCATCCTGGTC*	*18*			+			
			**18S-20nt-31x**					
18S#1	GCATATAACTACTGGCAGGA	20	+					
18S#2	TGTACTTAGACATGCATGGC	20	+					
18S#3	TGAGCCTTTTGCGGTTTCAC	20	+					
18S#4	CATGTCCCATAAGGTTCATG	20	+					
18S#5	ACGATCTTGCGATCGCTTGG	20	+					
18S#6	ACAAGACCATACGATCTGCA	20	+					
18S#7	ACAAGACCATACGATCTGCA	20	+					
18S#8	TTAGATGTGGTAGCCGTTTC	20	+					
18S#9	CCTCGGATATGAGTCCTGTA	20	+					
18S#10	TGCCCTCCAATTGGTCCTTG	20	+					

18S#11	ATACGCTATTGGAGCTGGAA	20	+					
18S#12	CACAAGTTCAACTACGAACG	20	+					
18S#13	CCACCGGTAATACGCTTACA	20	+					
18S#14	GTAATAGTACCGGCCCACAA	20	+					
18S#15	TTCAGGCATTTGAAGCCTGC	20	+					
18S#16	CAATGAAAGCAGAACAGAGG	20	+					
18S#17	TACTAATGCCCCCAAACTGC	20	+					
18S#18	TCTTTGGCAAATGCTTTCGC	20	+					
18S#19	TATGGTTAGAACTAGGGCGG	20	+					
18S#20	AAGTAGCTACACCCAATTGC	20	+					
18S#21	AGTCAAATTAAGCCGCAGGC	20	+					
18S#22	CCACGAACTAAGAACGGCCA	20	+					
18S#23	AAGCTTCAGCACCATAATCC	20	+					
18S#24	CAGGTACGGCTCCACTTACA	20	+					
18S#25	TGCTAGACGCAATTTGTCCA	20	+					
18S#26	ATCTAAGGGCATCACAGACC	20	+					
18S#27	CTCGGTCTAGGAAATACACG	20	+					
18S#28	TCAGTTCACAATCCCAAGCA	20	+					
18S#29	TGCGAGTTAATGACTCACAC	20	+					
18S#30	AAGGCGTCACAGTGATCACG	20	+					
18S#31	TACGGAAACCTTGTTACGAC	20	+					
			**osk-20nt-15x**	**osk-19nt-9x**	**osk17x**	**osk18x**	**osk24x**	**osk42x**
osk3'UTR#1	ATCGCGCAAATGCTTCAC	18				+	+	
osk3'UTR#2	TTAAGGGCAAGTGGCAGG	18			+		+	
osk3'UTR#3	ACGTGATCACCATCAATACA	20	+			+		
osk3'UTR#4	AGCTGTAAATTACGCCAGAA	20	+		+			
osk3'UTR#5	TGCTACAAACAAGCGCTTAG	20	+			+		
osk3'UTR#6	TCTGCAGCAGAGTGTAAGCA	20	+		+			
osk3'UTR#7	AATTTGCTTGAGCACATCAA	20	+			+		

osk3'UTR#8	GTTGATTTTGTGCAAGCGAA	20	+		+			
osk3'UTR#9	TTCCAAGTAAAGCAGTGCA	19		+		+		
osk3'UTR#10	TTACGGCCAAAATGCAGCA	19		+	+			
osk3'UTR#11	TGTATACGTACCACGCCCAC	20	+			+		
osk3'UTR#12	TGATACAGGAGCATGCCGAA	20	+		+			
osk3'UTR#13	GCGGAAAAGTTTGAAGAGAAG	21				+		
osk3'UTR#14	CTGCTTGCGCTTATTTTGCA	20	+		+			
osk3'UTR#15	CGAATTCCGTAAAAGCCGA	19		+		+		
oskCD#1	GATCCATCAGCGTAAATCG	19		+	+		+	+
oskCD#2	CCAACTTAATACTCCAGACTCG	22				+	+	+
oskCD#3	CCAGAACAGATAGGGTTCC	19		+	+		+	+
oskCD#4	TCGTTGATTAGACAGGAGTG	20	+			+	+	+
oskCD#5	ACAATAGTTGCCCAGCGG	18			+		+	+
**oskCD#6**	**TTTGTTAGAATCGGCACCAA**	20					+	+
oskCD#7	GCATATTGTGCATCTCCTTGA	21			+		+	+
oskCD#8	CTCGATCTGAACCAAAGGC	19		+		+	+	+
oskCD#9	ATAATGTCCACCGATCCGA	19		+	+		+	+
oskCD#10	GACGATGATCTGAGTACCC	19		+		+	+	+
oskCD#11	AGTCCGGATACACAAAGTCC	20					+	+
oskCD#12	CATTCGGGCGAGATATAGCA	20	+			+	+	+
oskCD#13	CATCGCCCATAAGCGGAAAG	20	+		+		+	+
oskCD#14	AGATAGGCATCGTAATCCGAG	21				+	+	+
oskCD#15	TCGTCAGCAGAGAATCGTTG	20	+		+		+	+
**oskCD#16**	**GTCATTTCGTGGCGTCTCT**	19		+		+	+	+
oskCD#17	GCTTTGGGTTCTGCAGCT	18			+		+	+
oskCD#18	GAGCCAAATTGATTGGTTCCTC	22				+	+	+
oskCD#19	GCTGTAGATGTTGATGGG	18			+		+	+
oskCD#20	GCATTTACGCTGGCTTGC	18				+	+	+

oskCD#21	AATTATCCTGGTAGCACCAG	20	+		+		+	+
oskCD#22	GTTTGAAGGGATTCTTCCAG	20	+			+	+	+
oskCD#23	AGGTGCTCGTGGTATGTTC	19						+
oskCD#24	TAGTCGCTGGTGCGCTCT	18						+
oskCD#25	AGCACCATATCCAGGAGG	18						+
oskCD#26	CGTTCTTCAGGCTCGCTT	18						+
oskCD#27	AAGATCCGCTTACCGGAC	18						+
oskCD#28	CTGCACTCAGCGGTCACA	18						+
oskCD#29	GGAATGGTCAGCAGGAAA	18						+
oskCD#30	CGTCACGTTGTCGTGCAG	18						+
oskCD#31	AAATGGATTGCCCGTCAG	18						+
oskCD#32	CTTGATGCTCGATATCGTGA	20						+
oskCD#33	TGGGCGTGGCTCAGCAATA	19						+
oskCD#34	CGCGCACCTCACTATCTA	18						+
oskCD#35	ATATTCCTCGCGCACGGA	18						+
oskCD#36	ATAGTTGCTCTCGATGATGG	20						+
oskCD#37	TGTTCTCGCTGGTGTTGC	18						+
oskCD#38	GTTGTAGGTGATTTCCTTGG	20						+
oskCD#39	TCTGAGTGGACGAGAAGAG	19						+
oskCD#40	GCTACGACTTGCAACTGC	18						+
oskCD#41	GAGTTCATGGGCCACCAA	18						+
oskCD#42	CTTCCACAACTCCGGCAA	18						+

* - Note, that EGFP#24 and EGFP#25 oligos are actually sense oligos reducing the effective hybridizing pool of the gfp23x probe set to 21 oligos.

Table S2: Probe synthesis calculator Excel sheet.
